# Supplementary material for: GENFIRE: A generalized Fourier iterative reconstruction algorithm for high-resolution 3D imaging
Source: Sci Rep. 2017 Sep 5;7:10409. doi: 10.1038/s41598-017-09847-1 (PMC5585178; doi:10.1038/s41598-017-09847-1)
Supplement: Supplementary file 1 — Supplementary Information [file 41598_2017_9847_MOESM1_ESM.pdf]

Supplementary Information

GENFIRE: A generalized Fourier iterative reconstruction algorithm for high-resolution 3D imaging

Alan Pryor, Jr., Yongsoo Yang, Arjun Rana, Marcus Gallagher-Jones, Jihan Zhou, Yuan Hung Lo, Georgian Melinte, Wah Chiu, Jose A. Rodriguez and Jianwei Miao

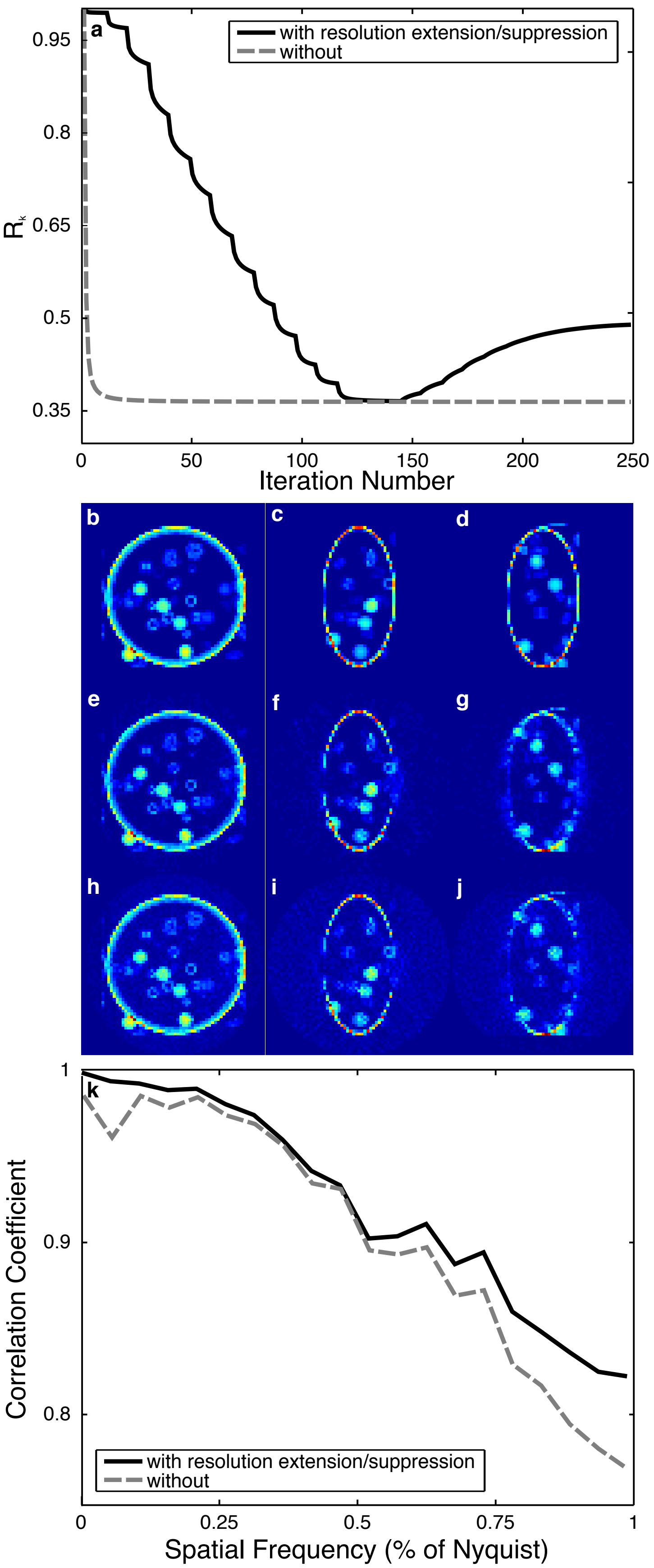

a
with resolution extension/suppression
without
R_k
0.95
0.8
0.65
0.5
0.35
0
50
100
150
200
250
Iteration Number
b
c
d
e
f
g
h
i
j
k
Correlation Coefficient
1
0.9
0.8
with resolution extension/suppression
without
0
0.25
0.5
0.75
1
Spatial Frequency (% of Nyquist)

Supplementary Figure 1. **a,** R_k as a function of the iteration number for GENFIRE with and without the use of the resolution extension/suppression technique. The abrupt dips in error over the first half of iterations correspond to events where the Fourier constraint is expanded. Projections of the model vesicle are shown along the three principle axes (**a-c**), with corresponding views for GENFIRE reconstructions with resolution extension/suppression **(e-g)** and without **(h-j)**. **k,** Fourier shell correlation between each reconstruction and the model indicating that GENFIRE with resolution extension produces a better reconstruction at all spatial frequencies, which is supported by the visual quality of the reconstruction depicted in **(b-j)**. Note that although the reconstruction is better across all spatial frequencies with resolution extension/suppression, as indicated by the Fourier shell correlation (**b-j**), the reciprocal error (R_k) is higher. The minimum error of R_k is achieved at the end of the extension step, and then begins to rise again during suppression. The reason for the increased error is that the true structure does not match the values of the (noisy) measurements. By enforcing only the less noisy low resolution values for the latter iterations, the structure is changed in a way that improves the quality of the reconstruction with respect to the model but not to the measurements. Therefore, although the reciprocal space error serves as a sign of convergence, it's value is not necessarily a measure of quality of reconstruction.

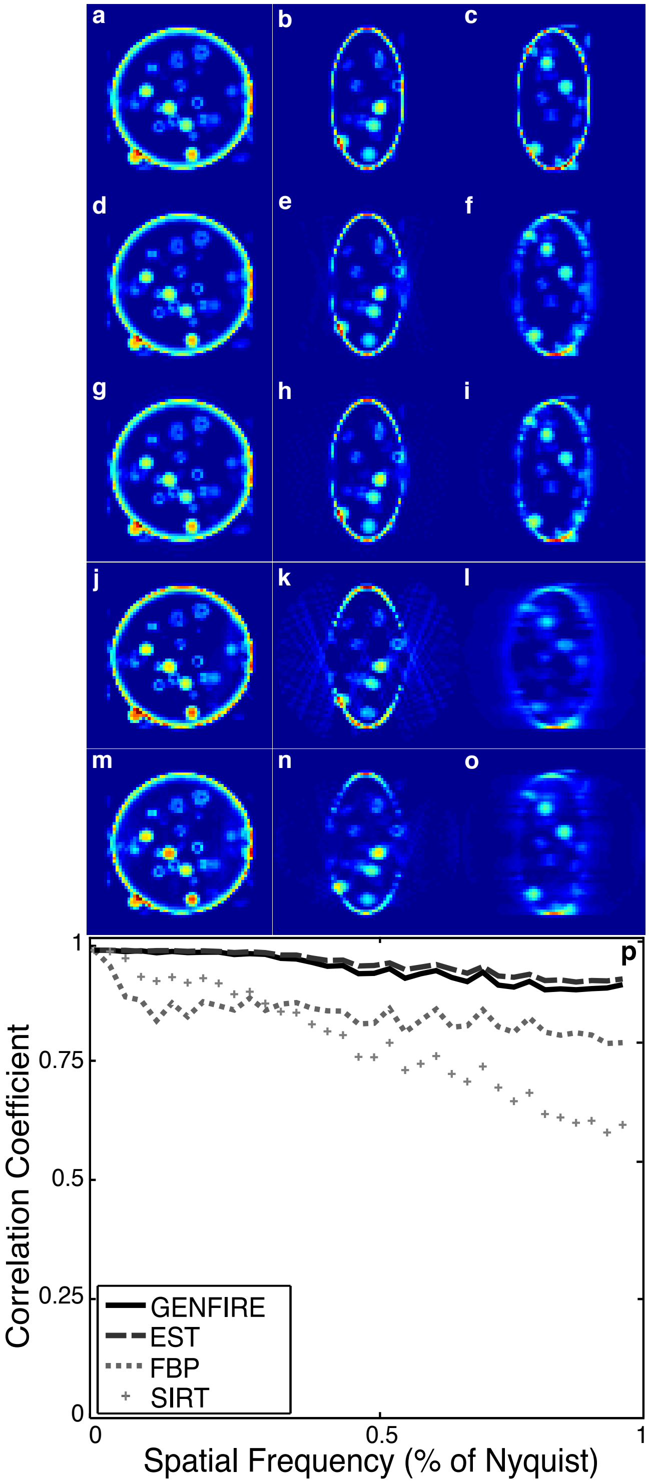

Supplementary Figure 2. Numerical simulations on the 3D reconstruction of a biological vesicle from 71 noise-free projections using GENFIRE, EST, FBP and SIRT. **a-c**, Three 10-voxel-thick central slices of the vesicle model in the XY, ZX and ZY planes, respectively. The corresponding three reconstructed slices with GENFIRE (**d-f**), EST (**g-i**), FBP (**j-l**), and SIRT (**m-o**). **p**, The FSC between the reconstructions and the model, showing that for noise-free data

with equal slope angles, EST produces slightly better results than GENFIRE as no interpolation is needed in EST.

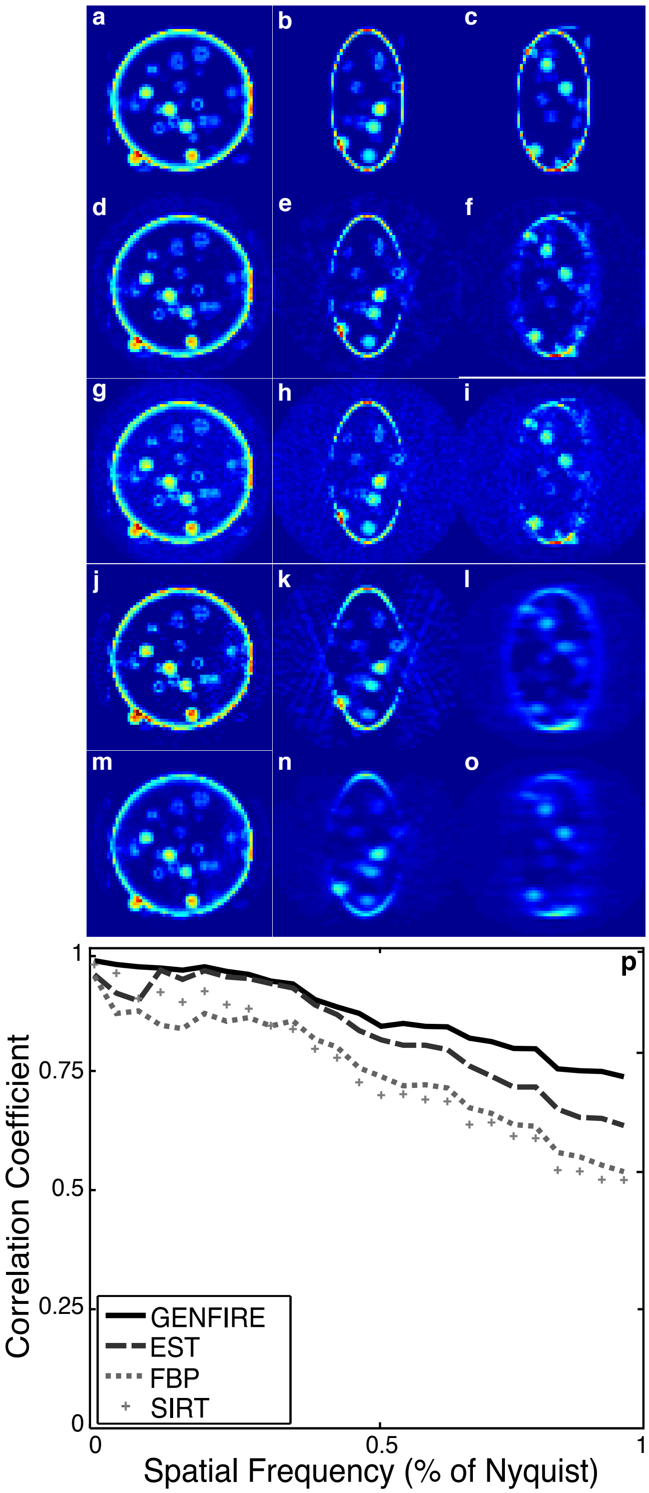

Supplementary Figure 3. Numerical simulations on the 3D reconstruction of a biological vesicle from 71 very noisy projections using GENFIRE, EST, FBP and SIRT. **a-c**, Three 10-voxel-thick central slices of the vesicle model in the XY, ZX and ZY planes, respectively. The corresponding three reconstructed slices with GENFIRE (**d-f**), EST (**g-i**), FBP (**j-l**), and SIRT (**m-o**). **p**, The FSC between the reconstructions and the model, showing that with very noisy projections, GENFIRE produces a more faithful reconstruction than other algorithms at all spatial frequencies.
